# Public health and democratic governance: COVID-19’s impact on South Africa’s democracy

**DOI:** 10.1007/s12286-023-00557-9

**Published:** 2023-03-07

**Authors:** Dirk Kotzé

**Affiliations:** Department of Political Sciences, University of South Africa, Pretoria, South Africa

**Keywords:** COVID-19 regulations, Quality of democracy, Separation of powers, South African democracy, State of disaster

## Abstract

The research is motivated by the need to determine the impact of South Africa’s COVID-19 regulations on its quality of democracy. It takes into account the interests of individual (liberal) rights in competition with the state’s interests of public security. Theoretical assumptions, based on classical democratic theories, which rely on the separation of powers and checks-and-balance principles, were used. The South African government architecture is assessed, especially in the context of accountability and oversight requirements. For this purpose, the relationship between the legislature and executive is most relevant. The South African government decided on a state of disaster to manage the pandemic, but it is contrasted with a state of emergency as the constitutional alternative. Its implementation, especially the institutional framework used for it, is analysed. The role of Parliament during the pandemic is used as an important test of the quality of democracy. The conclusions are that South Africa’s democratic principles did not degenerate during the pandemic, as concluded by Freedom House, but the pandemic’s major impact was on the quality of democracy. The state of disaster’s institutions, for example, were not those prescribed by legislation. Moreover, Parliament’s involvement in the state of disaster’s decision-making was limited. The 2021 local government election, on the other hand, was judged free and fair and its outcomes have been implemented without any public challenges. The main negative outcome is the public’s trust deficit in the ANC government’s use and abuse of pandemic regulations.

## Introduction

South Africa’s experience with the COVID-19 pandemic and in the manner in which its government managed it, raised concerns about its impact on democracy in the country. This was in line with concerns in several democratic states that their constitutional and political dispensations were directed towards more autocratic tendencies. During the pandemic, predictions were made that this trend could end in permanent regressions in democratic systems. The question therefore is: How did it affect South Africa as an example of a democratic state under African conditions? As a point of departure in trying to answer it, one can refer to Freedom House. For the year 2021 and based on individual scores for its political rights and civil liberties, South Africa was rated at 79/100. These scores remained the same as those before the pandemic (Freedom House 2021), which suggest that during the earlier part of the pandemic it did not cause democratic regression.

At the same time, in the short-term it is impossible to deny that these lockdowns restricted the functioning of democracy. Fears were expressed that they would become long-term practices of reducing the quality of democracy. South Africa as well as its neighbour Namibia, followed a very similar approach, and a gradual normalisation has taken place. The discussion here is directed at analysing the trend and reaching specific conclusions about it.

The discussion is organised in eight sections plus a conclusion at the end. First, it is necessary to articulate what the driving force of this discussion is and what the problem is that motivates it. A theoretical framework of the assumptions used in this discussion is thereafter presented. The fourth section introduces the South African government framework and its democratic characteristics. In the next section, the state of disaster as the preferred emergency response, is contrasted with a state of emergency, and their implications for democracy are discussed. In the sixth section, the state of disaster’s implementation is considered. It is followed by the institutional arrangements used by Government during the pandemic. In the last section, the focus is specifically on Parliament during the time of the pandemic.

## The problem for the research

The political implications of the Corona/COVID-19 pandemic are often presented by the media and in public opinion as a zero-sum choice between either a democratic or autocratic approach. This choice is too stark, because some states did not take a conscious political decision to use the opportunity to become more autocratic. Democracies in developing states (such as the Southern African states) have limited resources to deal with elaborate crises. The pandemic exposed the venerable elements of their economies, of limited social infrastructure or capital and of their ability to protect public health. It means that for the informal sectors in these societies to assist in providing public survival, restrictions on mobility or social interaction would be counter-productive. Therefore, in their cases, pandemic restrictions undermined the social fabric which supports a democratic infrastructure. Should these dynamics associated with the pandemic, be regarded as a process of de-democratisation?

A related problem is the relationship between individual (liberal) rights of freedom and the state’s communitarian public responsibilities. In a liberal democratic culture, steps taken by the public sector for the sake of public health security can be understood from an individual rights’ perspective as an intrusion into their rights. In a social democratic culture, on the other hand, those same steps can be seen as supportive of a democratic culture.

This serves as a background for a philosophical contestation. The theoretical and practical problems therefore are whether the pandemic regulations were seen as a challenge to liberal democracy or a necessary intervention to protect social democracy.

## The theoretical assumptions

The approach followed here is not to rely on one or a combination of theories as the lens of an analysis. The preference is instead to formulate a set of assumptions which can assist in structuring the discussion.

The discussion uses a theoretical framework which relies on the some of the best-known publications in the subject (Dahl 1972; Sartori 1987; Diamond and Morlino 2005; Schmitter and Karl 1991; Held 2006; Huntington 1991; Lijphart 1999; Przeworski et al. 2000).

The elements of that framework are the following:

1. Democracy is approached from either a procedural or a substantive point of view, or as a combination of the two. In this discussion, such a combination is preferred. The procedural aspects concentrate on constitutional arrangements (such as the separation of powers, rule of law, human rights, free and fair elections, and checks and balances on the executive), while substantive matters include socio-economic characteristics (such as the level of inequality, quality of life or economic opportunities) (Przeworski et al. 2000, p. 33).
2. The relationship between the legislative and executive authorities of the state relies on the principle of separation of powers and specific checks and balance arrangements, such as parliamentary oversight, motions of no confidence or budget approvals (Lijphart 1999, ch. 7). A too dominant executive poses risks for a democracy while a weak or inactive parliament jeopardises the democratic assumptions about public participation and representation.
3. The procedures of policy- and decision-making which are a derivative of the previous element. Decision-making in a democratic framework must be done in public, by public representatives and they must be accountable for it. The public administrators are responsible for implementation of the policies and for that they can have delegated powers, but the public representatives (executives) remain still responsible for it. Differences of opinion about the role of accountability and responsiveness in democracies were presented by Przeworski et al. (2000, p. 33) and Dahl (1972, p. 1).
4. Constitutional institutionalism emphasises the fact that decision-making and policy implementation must be done only by public institutions established by the constitution. Alternative or parallel, non-constitutional institutions are not compatible with a democratic state. Exceptional practices, such as a state of emergency, can justify temporary deviations from such institutional practices, but they are still determined by the state’s constitution and are not *ad hoc* arrangements.
5. Elections and electoral systems are also a key element. For the purpose of this discussion, the focus is on electoral principles such as that they must be free and fair (or at a high level of integrity) and be conducted regularly. Lijphart (1999, p. 7) followed the Schumpeterian approach and stated that democracy is present when a political system’s “most powerful collective decision makers are selected through fair, honest, and periodic elections in which candidates freely compete for votes in which virtually all the adult population is eligible to vote”.
6. Quality of democracy: Diamond and Morlino (2005, p. xi) define a quality democracy “to be one that provides its citizens a high degree of freedom, political equality, and popular control over public policies and policy makers through the legitimate and lawful functioning of stable institutions”. They identified eight dimensions which determine democratic quality, namely the rule of law, participation, competition, vertical and horizontal accountability, respect for civil and political freedoms, progressive implementation of greater political and socio-economic equality, and responsiveness (Diamond and Morlino 2005, p. xii).

These six elements provide the frame of reference for our use of “democracy” as a concept and analytical yardstick in this discussion.

Most of the existing theories do not give specific attention to exceptional circumstances such as natural disasters, global economic crises, periods of war or health pandemics. Except for wars, these situations (including the COVID-19 pandemic) are not caused by political players but are beyond their control. They are therefore not intentional but a form of *force majeure* or an act of God. They are also quite often temporary in nature and produce a crisis for which states seldom are prepared. Quick responses which are not the products of meticulous planning, are most of the time required.

Wars or political emergencies in the context of democratic theory received more attention than natural or health disasters. An example is the work of Carl Schmitt and his “state of exception” doctrine to justify undemocratic emergency powers. His contribution is an example of working outside a democratic framework and therefore not directly relevant for this discussion. A more relevant presentation is by Zwitter (2012) who concentrated on the use of a state of emergency in democracies. The focus is exclusively on the rule of law in a state of emergency. It therefore deals with only one aspect of a democracy and does not present a complete theory of democracy during emergencies. Afsahi et al. (2020) edited a special volume of *Democratic Theory* on democracy in a global emergency. The authors identified five lessons learnt from the COVID-19 pandemic. According to them, the pandemic can be characterised as “the hour of the executive” and the demise of parliaments. At the same time, they concluded that in states where democracy was already under threat, the pandemic accelerated their democratic erosion while stronger democracies were less affected by it (Afsahi et al. 2020, p. vii).

We have to accept that theories in general don’t concentrate on the exceptions—the purpose of theories is to identify general trends, correlations or relationships. The majority of democratic theories, therefore, do not accommodate such interruptions which are not part of political agendas. Regressions in democratic dispensations are presented as either counter-waves (Huntington 1991), developments towards competitive authoritarianism (Levitsky and Way 2002), illiberal democracy (Zakaria 1997) or de-democratisation (Freeman 2017), all of which are regime changes. The possibility of externally imposed factors which negatively affect a democracy, but which can later disappear (like pandemics) or be reconstructed (like natural disasters and economies), are not necessarily part of such theories.

In view of these contentions, the premise used here is that the governments’ interventions such as pandemic regulations, serve as temporary limitations presumably with a direct, negative impact on the quality of the democracies, but not necessarily on the existential democratic components, such as the basic democratic values, institutions and behaviour of the main role-players. The democratic system’s constitutional foundation also remains intact.

A conventional interpretation of the limitations placed on democratic rights (by these exceptional circumstances) will most possibly conclude that it is a form of democratic erosion or regression and approaching autocratic rule. If the exceptional circumstances are accepted as constituting a *sui generis* situation for only a limited period of time, it implies that the main focus should not be on a regression of the existential components of the democracy and their *consolidation*, but could it find better expression in an assessment of the *quality* of the democracy. Only when it is clear that these limitations are not for an interim period only but for the long-term, and that they are motivated to replacing democratic features with new, autocratic practices, then democratic regression is at stake.

The next step is to determine theoretical assumptions which could inform our understanding of the nature of such limitations and our assessment of whether they are fundamentally undemocratic or only temporary in nature. For this purpose, the starting point is to use the theoretical principles of the separation of powers.

It is assumed that the Executive have the strongest propensity to concentrate state power in itself. The separation of powers is therefore firstly used to prevent such usurpation of power. Legislatures are primarily responsible for this task but supported by the Judiciary. If exceptional circumstances paralyse the Legislatures, democracy would be under pressure. The Judiciary would then be the only state authority which could counter the executive power. If the Judiciary is unable to secure it, democracy will be in a crisis.

The second theoretical assumption depends on the relationship between the political and civil societies. Though without any state authority, an independent and institutionalised civil society can be an effective watchdog of the Executive. They have a number of options. One is to use courts as a platform for litigation against the Executive (if the Judiciary is independent enough). The other one is public means such as the media, churches or public demonstrations. Prolonged and widespread public defiance would be an indicator that the Judiciary on its own is not effective as a watchdog and that the public has lost confidence in the checks and balances typical of democratic systems. It would mean that the democratic dispensation has collapsed and regime change is imminent.

Guided by all the theoretical considerations, a set of focus areas should be identified as a matrix for analysing the interaction between the pandemic dynamics and the South African government’s political management of it. The first was whether “the hour of the executive” materialised in South Africa and how it affected the democratic architecture of the country. The second is its counter-focus, namely Parliament’s role in the emergency. Thirdly, civil society’s societal role and interaction with political society as a buffer against democratic decline has to be aligned to the emergency dynamics. It was not paralysed by the emergency regulations but used innovative methods in gathering information, providing information to the public, using court cases and acting as a public watchdog. The fourth focus area in the suggested matrix are the substantive elements of democracy which can present the socio-economic dimensions of the pandemic regulations. Increased unemployment, closure of small and medium-size businesses, an increase in food prices are socio-economic implications of the restrictions imposed by the pandemic regulations in many states. Without an in-depth statistical analysis of the South African economy during the pandemic, President Ramaphosa’s reference to them in his monthly public broadcasts already confirmed their relevance for managing the pandemic. Finally, such an analysis has to reach conclusions on whether and how South Africa’s democratic consolidation had been affected by the pandemic’s political management. It is important to relate it also to South Africa’s quality of democracy. Diamond and Morlino’s eight dimensions of democratic quality (mentioned earlier) are the guiding principle to clarify this point.

Regulatory restrictions are not *per se* sufficient indication that democracy has been eroded or limited. The South African constitution allows for restrictions of rights even in times of normality. Section 36 in the Constitution is a demonstration of how limitations can be accommodated in a constitutional context. Its formulation is: “The rights in the Bill of Rights may be limited only in terms of a law of general application to the extent that the limitation is reasonable and justifiable in an open and democratic society based on human dignity, equality and freedom, taking into all relevant factors”.

The implication of this formulation is that democratic rights (including human rights) can be limited and still meet the democratic standard. It is useful for our theoretical contention that in some instances and for some periods, limitations could be reconciled with democratic principles. Objective standards, however, are absent here, because it depends on what is acceptable in other open and democratic societies and that might also differ from case to case.

Also informative for the discussion, is Section 37 which deals with a state of emergency in South Africa. A state of emergency is a relevant option in exceptional cases, as discussed earlier and therefore it is worth noting it. It is accompanied by a table of non-derogable rights which cannot be limited in exceptional cases, and which is the opposite of Section 36.

## The South African government framework and democratic characteristics

In order to evaluate the nature, democratic or otherwise, of government activities during the COVID-19 pandemic in South Africa, the constitutional framework of government has to be presented in summarised form. It is discussed here in the context of the separation of powers framework, which has a very particular application in South Africa.

The macro-framework is that the South African state is a republic, although it includes traditional leadership and monarchies. Though they have powers of their own, they are subject to the Constitution’s authority and therefore the President, Parliament and the judiciary.

Government is divided into three spheres: national, provincial and local. The legislatures in all three spheres are directly elected every five years by South African citizens of 18 years and older (SA Constitution 1996, S. 46). Only the second house of Parliament, the National Council of Provinces (NCOP) is not directly elected. Its ten members per province are indirectly elected by the provincial legislatures. The National Assembly has the right to remove the President by impeachment (SA Constitution 1996, S. 89) or the whole Executive (including the President) by a motion of no confidence (SA Constitution 1996, S. 102).

The President is the head of the Executive (SA Constitution 1996, S. 85). The National Assembly elects the President after a general election or when a vacancy arises, for a term of five years and a maximum of two terms (SA Constitution 1996, S. 87; Kotzé 2019). It is therefore not a directly elected position. The President does not remain a member of Parliament but the newly elected incumbent must immediately resign as a member of Parliament. It is therefore not exactly similar to a prime minister. The President appoints the Deputy President and the Ministers. All of them, except for two, must be members of the National Assembly. All the Cabinet members can be removed by the President or by a parliamentary motion of no confidence.

The relationships for ensuring accountability and oversight in terms of the separation of powers framework include characteristics of both the presidential and parliamentary systems. The Deputy President and the Ministers, according to the Constitution (S. 91 (2)) are responsible for the executive powers and functions assigned to them by the President. They are collectively and individually accountable to the President and Parliament for these powers. The Constitution (S. 55 (2)) also requires that the National Assembly must provide mechanisms for the executive organs of state in the national sphere to account to it.

This is the constitutional framework of government within which the COVID-19 pandemic had to be managed. Later, the Constitution’s provisions regarding a state of emergency (S. 37) will also be presented.

Different government systems require different processes to meet the essential requirements of a democracy. At the same time, the principles of democracy remain the same, irrespective of the government system. The South African system is neither presidential/semi-presidential nor parliamentary. The President’s position combines characteristics of a both a prime minister/premier/chancellor and an executive president. This hybrid shares similarities with the systems in Botswana, Angola and Pacific islands. It includes exclusive presidential powers, presidential powers shared with ministers, parliamentary powers (such as selecting of candidates for appointments made by the President) and oversight over the Executive. This mixture is partly the result of the fact that the majority of Cabinet members are also members of Parliament. Even more unique, is the fact that the Deputy President is the Leader of Government Business in the National Assembly, not as a “prime minister” but as the presidential representative to coordinate the work of the ministers in Parliament (SA Constitution 1996, S. 91 (4)). This might look like a semi-presidential arrangement, but it is not, because the Cabinet is directly appointed by, and responsible to, the President (Kotzé 2019).

For the purpose of the COVID-19 pandemic, the quality of the relationship between the legislature and the executive is of paramount importance. Could the essential elements of this relationship continue during the pandemic? What were these essential elements? One can assume that it was not necessarily possible for the *form* of these elements to remain the same as during normal times, but the *substance* of these elements should not become derogated.

## A state of emergency or a state of disaster

In March 2020 it was already clear that COVID-19 was a global phenomenon and therefore a pandemic. Its impact on populations, its spread and possible mutations were unpredictable. Developed economies with an established healthcare infrastructure expected to be better prepared for it than developing countries. They required emergency interventions. South Africa has a bifurcated healthcare system. One part is a very sophisticated private healthcare infrastructure, and the other part is a poorly maintained public healthcare system. Most of the low-income population depends on the public sector infrastructure. The question was how to mobilise and use the available resources most effectively under these unpredictable conditions. The government decided on declaring a state of disaster.

The criticism of how the pandemic was managed and the negative perceptions that it harmed South Africa’s democracy, are mainly concerned with the state of disaster. It therefore deserves close attention as the main policy and institutional arrangement for managing the pandemic.

The South African constitution (1996) provides for a state of emergency (S. 37) but not for a state of disaster. Both emergency arrangements are, however, sanctioned by national legislation: the State of Emergency Act, 64 of 1997, and the Disaster Management Act (DMA), 57 of 2002. A comparison between the two can give us an understanding of what the options were, why the state of disaster became the preferred option and what are the implications.

The Constitution in S. 37 (1) determines that a state of emergency can be declared by the President only when “the life of the nation is threatened by war, invasion, general insurrection, disorder, natural disaster or other public emergency and a declaration is necessary to restore peace and order”. A state of disaster, on the other hand, can be declared by the earmarked Minister if the existing legislation and contingency arrangements do not adequately provide for the national executive to deal effectively with a disaster (Disaster Management Act 2002, S. 27). The main difference between the two is not in terms of the circumstances they are designed for, but the fact that the President takes charge of emergencies and the Minister (of Cooperative Governance and Traditional Affairs—COGTA) of disasters. Though not specified in the Act, the Disaster Management Act (DMA) appears to be tailor-made for natural disasters like floods, fires or earthquakes, while a state of emergency extends it further into political and other nation-wide crises. The question remains of which one is best suited for a public health crisis like the COVID-19 pandemic.

The next comparative dimension is how the two emergency responses are institutionally formalised and the conditions for their application. The President can declare a state of emergency but within 21 days the National Assembly must resolve to extend it, otherwise it is automatically terminated. It may not be extended for more than three months at a time. The first extension must be approved by a majority of 50% + 1of the members of the National Assembly (It means 201 of the 400 members, irrespective how many of them are attending the session.). The second and further extensions must be approved by at least 60% of the members (i.e., 240). S. 37 (2) of the Constitution prescribes that such a resolution can be adopted only after a public debate in the National Assembly. The emphasis is therefore on the fact that because it is an extraordinary arrangement, Parliament must be fully engaged in the decision-making, and it cannot be left in the hands of the Executive. The checks-and-balances function of the separation of powers is clearly visible in this logic.

In the case of a state of disaster, it is declared by the earmarked Minister (of COGTA). Its renewal does not require parliamentary approval or a public debate. It lapses after three months, similar to the state of emergency. If it has to be extended, it could be done for one month at a time by the Minister (Disaster Management Act 2002, S. 27 (4)). In practice, it involved a process initiated by President Ramaphosa by consulting various stakeholders before a final decision was taken by the Cabinet. Usually, he also announced the renewal decision on a live TV broadcast every month, which he called a “family meeting”.

The most important difference between the two emergency responses is the role played by Parliament in their oversight and legitimisation. The fact that the Executive-dominated state of disaster was chosen by the South African government, created fault-lines in its implementation and made democratic risks possible.

A controversial component of South Africa’s state of disaster was the ministerial regulations published on a monthly basis in the official *Government Gazette* after the public announcement by President Ramaphosa. The state of emergency’s stipulations in the Constitution on regulations are in much more detail than those in the state of disaster’s legislation. They concentrate in particular on legislation or regulations which may derogate from the Constitution’s Bill of Rights. Such derogation, according to S. 37 (4), will be acceptable only if it is consistent with South Africa’s obligations under the rules of international law on a state of emergency, and if they are announced in public. This non-derogatory emphasis also means that legislation or regulations would not be allowed to indemnify the state or any person in respect of any unlawful act. Limitations on the human rights of equality, human dignity, life, freedom and security of the person, slavery and servitude, children’s rights and arrested, detained and accused persons will also not be permissible (SA Constitution 1996, S. 37 (5)).

The state of disaster legislation does not include comparable non-derogatory stipulations. Instead, the over-arching and stricter constitutional requirement applies to it that at all times and in all respects that it must be constitutional. The admissible restrictions are the limitation principle in S. 36. This section states that the rights in the Bill of Rights can be limited “to the extent that the limitation is reasonable and justifiable in an open and democratic society based on human dignity, equality and freedom”. Five factors are also listed which can serve as additional criteria. Comparing all the considerations, both emergency options have their own positive elements. The constitutional option of a state of emergency includes more guarantees for democracy than the legislative framework of a state of disaster. A state of disaster, on the other hand, is ruled by a constitutional dispensation applicable in normal circumstances and any legitimate or legal derogation of rights will be admissible only in terms of those norms.

## Implementation of the national state of disaster

The Minister of COGTA, Dr Nkosazana Dlamini Zuma, on 15 March 2020 declared a national state of disaster in the *Government Gazette* (Dlamini Zuma 2020). A set of regulations followed the declaration which included restrictions on international travelling, prohibition of gatherings of more than 100 persons, schools, universities and other educational institutions were closed, time restrictions were placed on selling of alcoholic and tobacco products, and a curfew was introduced.

Soon thereafter, on 23 March 2020, President Ramaphosa addressed the South African public on a live TV broadcast. His message was that in response to a dramatic increase in infections within a week, the National Coronavirus Command Council “has decided to enforce a nation-wide lockdown of 21 days” from 26 March (Ramaphosa 2020, p. 2–3). A range of categories of emergency workers and essential services were exempted from the instruction to stay at home. Exceptions such as buying of food and medicine, or seeking medical care, were allowed. All other businesses were closed. Both South Africans returning home as well as international travellers from high-risk states, had to remain in quarantine for 14 days. President Ramaphosa also announced that he would deploy the SA National Defence Force to assist the SA Police Service in enforcing these regulations (Ramaphosa 2020, p. 3–5).

A package of relief measures was also announced in this speech. It included the new Solidarity Fund, managed by the private sector, to work in partnership with the public sector. Steps were announced to prevent price hikes; arrangements were made with the retail sector to secure a supply of household goods; and employees in distress could be assisted by the Temporary Employee Relief Scheme. The South African Reserve Bank also decided to reduce the repo rate by 100 basis points (Ramaphosa 2020, p. 5–8). This was the beginning of a process of imposing restrictions on a monthly basis—every time when the state of disaster was renewed. A scale of levels 1–5 was announced to determine the severity of the situation and the appropriate restrictions for each one (South African Government n.d.). Monthly evaluations done by the Health Minister’s Ministerial Advisory Committee determined the appropriate level of restrictions.

A summary of the restrictions as well as the relief measures are presented here in order to develop an appreciation of their scope. They are a key element of the argument that the pandemic had had a direct, negative impact on the quality of democracy in South Africa. The experience of the first three weeks was the worst during the two years until 2022 when the state of disaster was terminated. During the two years, five “waves” of infections were experienced in South Africa. The regulations which caused the most serious public outcry by civil society organisations for their “irrational or unjustified nature” (Staff writer 2021), were introduced during December 2020/January 2021. At the peak of the summer holidays, the beaches were closed, trade in tobacco products were stopped and alcoholic products were severely limited. International travelling was prohibited and the tourism industry almost closed down (Ndou 2020). Court cases by political parties, NGOs and other civil society organisations challenged these regulations, and in some cases were successful.

This situation was managed by a set of government institutions which also deserve attention. During the 3‑week lockdown, Parliament closed and most of the government institutions could not function in a normal manner. A climate of abnormality existed, and the question is how it affected the essential democratic elements. Up to now policy- and regulatory matters received most of the attention. Their impact on democratic practices (including the human rights dispensation) is one aspect of a democratic analysis. The other is the impact of institutions on it, and in particular the decision-making processes. The next section deals with this.

## The institutional architecture of the pandemic management

On 30 June 2022, the Office of the President release a report prepared in their personal capacity by various professional experts and coordinated by the Department of Planning, Monitoring and Evaluation, the Government Technical Advisory Centre and the National Research Foundation. This *South Africa Covid-19 Country Report* (Presidency of South Africa 2021) attended, amongst several topics, to the legislative frameworks used by the Government during the pandemic and the institutions or management systems utilised during this time. The conclusions are generally critical, such as that the Government did not apply appropriately the Disaster Management Act and its structures. It also concluded that using institutions of the security cluster during the pandemic was inappropriate for a health crisis (Presidency of South Africa 2021, p. 117–139; Du Plessis 2022).

The first edition of the report of more than 700 pages is the first comprehensive public assessment of the pandemic’s management. It sets the scene for more evaluations or analyses of this aspect, and hence some of them will be addressed here.

The Disaster Management Act (DMA) was the Act in terms of which the state of disaster was declared and renewed on a monthly basis. The regulations gazetted by the Minister of COGTA were also done on the basis of this legislation’s authority. One can therefore presume that the institutions and procedures embodied in this Act constitute the institutional framework of the pandemic’s management.

The Act, in S. 4, provides for the Intergovernmental Committee on Disaster Management. The President must establish it, consisting of ministers responsible for the various functions of disaster management, members of the provincial cabinets (Executive Councils—MECs) in the provinces affected by the disaster, and members of local government. The Minister (of COGTA) chairs the committee, which is accountable to, and advises, the Cabinet.

The second institution is the National Disaster Management Centre. It must promote an integrated and coordinated disaster management system, develop communication links with the disaster management role-players, be responsible for information gathering, give advice and guidance, and develop disaster management plans and strategies. It is a permanent centre constituted of professional security and disaster management professionals (Disaster Management Act 2002, S. 8).

The third institution is the National Disaster Management Advisory Forum (Disaster Management Act 2002, S. 5). The Minister must establish the forum consisting of the Head of the National Centre (as the chairperson), senior representatives of the ministers and MECs on the Intergovernmental Committee, municipal officials and a range of civil society role-players (including organised business, organised labour, the insurance industry, organised agriculture, traditional leaders and several others). The Forum has to make recommendations to the Intergovernmental Committee, any organ of state or other relevant bodies. One can conclude that this is meant to be a consultative body which could be used to involve a wide range of society’s interest groups.

The astonishing fact is that these institutions were not activated by Government during the pandemic and instead other institutions emerged.

Soon after the state of disaster was declared, President Ramaphosa started mentioning the National COVID-19 (or Coronavirus) Command Council, consisting of 19 ministers relevant for the pandemic management, and the departmental heads of these departments (including the defence force and the police service), and chaired by him. This council did not have any legislative origin. President Ramaphosa’s public references to it included phrases like “decisions by the Council”. Challenges by opposition parties that this is an unconstitutional body with no decision-making authority, is probably the reason why President Ramaphosa changed it to the Council being an advisory body of Cabinet and that Cabinet takes the final decisions. The Council was replicated also at provincial and local levels. One can conclude that the Command Council replaced the Intergovernmental Committee on Disaster Management without any formal government decision or announcement.

President Ramaphosa also utilised another institution for consultation purposes: the Presidential Coordinating Council (PCC), co-chaired by him and the Deputy President and consisting of ministers, the provincial premiers and the mayors of metropolitan councils (The Presidency 2020). The PCC was established in terms of the Intergovernmental Relations Framework Act, 2005, and in this respect differs from the Command Council, without a legislative base. Arguably, the Command Council and the PCC took the place of the Intergovernmental Committee in the management structure.

Every month before the state of disaster’s term expired and before its renewal the same chain of events involved these institutions. It commenced with a meeting of the National Command Council that evaluated the latest situation and decided on the regulations required for the coming month. It was followed by a PCC meeting, then followed by presidential consultations with political party, traditional and religious leaders, business leaders or in some instances the National Economic Development and Labour Council (Nedlac), which is a corporatist forum of organised business and labour, and government representatives. The final step was a Cabinet meeting to finalise the decision.

The DMA’s Advisory Form did not feature in these processes. Instead, the Minister of Health established his Ministerial Advisory Committee (MAC) on COVID-19. The Minister has the authority in terms of the national Health Act to appoint advisory and technical committees. Their composition, functions and terms of reference must appear in a notice in the *Government Gazette*. Three MACs were established during the pandemic period. The first one became best-known: the MAC on COVID-19, consisting of 51 medical doctors and medical science academics, and established immediately after the state of disaster. No *Government Gazette* notice appeared of its formation and therefore its terms of reference remained unclear. The second MAC on coronavirus vaccines appeared months later. It was followed by the Multisector MAC on social and behavioural change (Singh 2020; Makou 2020).

The MACs fulfilled different functions during the two years of the pandemic. Being primarily a scientific body, their advice to the Health Minister was often also used in the Command Council. President Ramaphosa referred to it in his public statements as an approach to decision-making which is based on scientific facts and not political considerations. It was used as a means of legitimising often unpopular announcements about curfews, wearing of masks or limitations on large gatherings. This phenomenon assumed an interesting public dimension when differences inside the MAC spilled over into public statements by some of the medical experts, or accusations by them that their advice was not followed when the Cabinet took its final decisions (Human and Geffen 2020). It the minds of some political parties and the public, they became too influential in the decisions and therefore calls were made that their scientific advice be made public, but it never happened.

Though the National Disaster Management Centre is permanently in existence, it did not play the role set out in the DMA during the pandemic. In its place, the National Joint Operational and Intelligence Structure (NatJoints) became a prominent element in the decision-making process. As a standing body, it consists of the SA Police Service, SA Defence Force, National Prosecuting Authority, Department of Health, the National Intelligence Coordinating Committee (NICOC) and other government institutions, depending on what the security issue is. NatJoints monitored on a daily basis the infection and vaccination rates, new trends in the pandemic, possible risks in terms of governance problems, food security issues, economic risks or social instability. During the pandemic, it submitted regular reports to the National Command Council and was instrumental in formulating the regulations every month for the state of disaster (It could be said that their role competed with those of the MAC in some instances.). The *South Africa Covid-19 Country Report* observed that NatJoints, as part of the security and intelligence cluster, was not the most appropriate institution for this purpose and the DMA’s bodies, such as the Centre, could have been more apposite (Du Plessis 2022). A conventional intelligence and risk analysis is expected to result in a product more biased towards security considerations and not necessarily managing of health or social problems. It is arguably part of the explanation why some of the regulations were overly harsh and regarded in public as irrational.

A key difference between the institutional framework of the DMA and the one used by President Ramaphosa, is that the DMA framework is primary in the hands of the Minister of COGTA, while the Ramaphosa framework was controlled by himself. It can be interpreted as Ramaphosa’s institutionalisation of presidential power. He has been accused since assuming the presidential role of being indecisive and insecure. Now the pandemic provided an opportunity for him to enhance his prominence. It can also be linked to his party, the African National Congress (ANC)’s internal politics. The COGTA minister, Dr Dlamini Zuma, was his main opponent in the ANC’s 2017 presidential election. As a former wife of President Zuma, she was the candidate of his faction in the leadership election. President Ramaphosa arguably wanted to deny her the centre-stage during the pandemic. By appearing on national TV every month for his “family meeting” with the South African public to assess the pandemic and announce the regulations for the coming month, it gave him the opportunity to communicate directly with the public. It increased his popularity.

The question is: how did this institutional approach affect the democratic quality during the pandemic? Firstly, the DMA was used to authorise the state of disaster and the pandemic regulations, but the Government did not use its institutions. The preferred institutions, all of them except for the Command Council, were “co-opted” into the management framework. It resulted in an alternative for the DMA framework. That is not necessarily undemocratic but the need for such an approach was never publicly explained. The democratic need for public accountability was not upheld in this case. Involvement of NatJoints, in particular, was not kept secret from the public but they were not appropriate for these circumstances. Their presence introduced a security approach to a health crisis, which allowed the police and military to grow in prominence. Also noticeable is that public interaction with these institutions was limited. President Ramaphosa did use consultation which included political, religious and traditional leaders and Nedlac, but it was limited in scope. As a result, in a number of court cases the constitutionality of the DMA and of the COVID-19 Command Council were challenged. In several instances, specific regulations were also successfully challenged in court. Though the role of the MACs was commendable, their emphasis on scientific evidence concentrated on the rationality of decisions and also included an elitist component of privileged access to information not known to the public in general. Finally, the separation of powers requirements were inhibited by the reduced role of Parliament during the two years. This is now briefly considered.

## The role of parliament in the pandemic governance

Parliament’s relationship with the pandemic and the state of disaster was determined by several factors. The first one is that in the discussion of a state of emergency and a state of disaster in South Africa, the different roles of Parliament emerged as one of the main distinguishing characteristics between them. In a state of disaster dispensation, Parliament plays a very limited role. The second factor is that the pandemic regulations also affected the activities of Parliament itself. The third factor is the separation of powers principle as well as the checks-and-balance role Parliament plays in this principle. Parliament’s role in the political system during the pandemic is one of the key considerations in determining the pandemic’s impact on democratic quality.

The first indicator of discontent about the Parliament’s role in principle in the state of disaster, was when the Freedom Front Plus (FF+) opposition party tabled on 19 February 2021 the Disaster Management Amendment Bill (B2-2021) in the National Assembly. The purpose of the Bill was to amend the duration of a state of disaster to be the same as that of the state of emergency. The amendment also proposed to change the decision-makers so that only the National Assembly may extend the state of disaster, and the required majorities for such decisions should be similar to those of a state of emergency. Like a state of emergency, another amendment said that extensions can be made only after a public debate in the National Assembly. The purpose was clearly to bring the state of disaster in line with the state of emergency in terms of parliamentary powers, but the amendment was rejected by the National Assembly in June 2022 (Parliament/Bills n.d.).

Another reflection on the role of Parliament, is the fact that the initial lockdown regulations during the first three weeks applied to Parliament also and therefore it was closed with no activities at all. The parliamentary management developed immediately an online communication platform for members of Parliament and all the parliamentary committees. From personal observation, for most of 2020, all parliamentary activities were conducted by online means. That included plenary sessions, committee meetings and the administrative functions of the Speaker and other office-bearers. During 2021, a hybrid system was introduced, combining online and in-person participation in Parliament. That was still regulated by the general pandemic regulations of social distancing and the maximum number of participants at big events. One of the parliamentary procedures affected by these regulations, was voting in the National Assembly. Before the pandemic, an electronic voting system was used for voting by hand in public. The online system did not enable a similar procedure and members had to inform their party Whips how they vote on an issue. During the vote, the Whips report in the session their party’s number of votes to the parliamentary presiding officer. It does not affect the public nature of the voting process, but it could inhibit members from taking an independent stance in the vote. The South African parliamentary tradition is dominated by parties and their parliamentary caucuses. Back-bencher voting therefore seldom occurs, but the principle remains that it should be possible. The Whips’ direct involvement in the online voting amounts to gatekeeping, which is undesirable.

In June 2021, the Speaker of the National Assembly, Thandi Modise (2021), reported on parliamentary activities for the 2020/2021 year. This was the first year of the pandemic and the period which most affected Parliament. She reported that during this period, Parliament and its committees produced 408 reports, conducted 40 oversight visits, scheduled 60 public hearings and convened 48 virtual meetings. These are less than in a normal year but probably more than the public perception of Parliament’s performance. Emphasis was also placed on parliamentary communication with the public. Modise (2021) summarised it as follows:

‘With the outbreak of [the] Coronavirus, we had to increase and enhance our communication channels. Our meetings as well as other business of the Houses have been broadcast live, streamed on channels like YouTube, Twitter, and Facebook. We have an additional platform called iono-fm which enables stations and the public to access recordings of out sittings. We launched Parliament TV for live broadcast.’

Plenary sessions, decision-making and communication were not Parliament’s main problems. The more complicated one was the parliamentary oversight role. Though portfolio committees were operational, interaction of their members with ministers or senior officials were often hampered by technical problems, while the intimate nature of interactions were absent. It reduced the quality of oversight. Moreover, the online style of debating reduced the opposition’s impact on the governing party (ANC). It also reduced the parties’ visibility in the media (especially TV) and their ability to register their presence in important public issues. Especially during the first part of the pandemic, Government dominated decision-making and communication with the public on related matters. Political parties, including the ANC, took a backseat in this regard. While the pandemic was publicly accepted as a serious national crisis, the Government’s dominance was accepted as a national imperative, but after a few months that sentiment dissipated.

These trends resemble the expectations about government-opposition relations identified by Louwerse et al. (2021, p. 1025–1026). For these authors, from a normative democratic perspective, in times of crises these relations have contrasting implications. On the one hand, government/opposition consensus on crisis measures in the beginning of a crisis, increases the public legitimacy of the initial steps in the pandemic. On the other hand, criticism by the opposition serves as a catalyst for public debates, which is indispensable for democracy. Louwerse et al. (2021, p. 1026) expected that as the COVID-19 pandemic evolved, the nature of the crisis changes from a critical public health crisis to a combination of a public health, social and economic crisis. In this process, the parties’ sentiment towards government also changes.

The implicit limitations on the South African parliamentary processes during the pandemic definitely reduced the quality of South Africa’s democracy, both in terms of institutional autonomy (i.e., the separation of powers), oversight over the Executive and decision-making regarding the state of disaster.

## Conclusion

The conclusions are presented in terms of the five factors or variables presented in the theoretical section as probable determinants of democratic regression.

The first one is the duration of executive limitations, based on the assumption that the longer they are in operation, the more the limitations can become institutionalised and the more of a negative impact they can have. President Ramaphosa terminated the state of disaster on 4 April 2022 (Ramaphosa 2022), while all the remaining regulations came to an end on 23 June 2022. No formal institutions, regulations or arrangements remain in place which can continue with pandemic emergency functions. Institutionally, all executive actions have been concluded. One attempt was made to create an alternative for future pandemics. After the end of the state of disaster, the Minister of Health published draft regulations for health crises based on the Health Act for public comments. The public response was so negative that they were not approved. The end-result is that the executive role during the pandemic could not be entrenched and therefore it did not have a lasting effect on South Africa’s democracy.

The role of Parliament, as the second factor, is more complex. The choice of a state of disaster instead of a state of emergency certainly curtailed Parliament’s influence on it. The fact that Parliament was itself subjected to the disaster regulations also limited its activities. Moreover, opposition parties had less public exposure and fewer opportunities to register this presence during the pandemic. The pandemic’s management, especially in its earlier stages, was dominated by Government and political parties—including the ruling ANC—played a minor role in it. Parliament’s downsized position during the pandemic reduced the quality of democracy. A major fire on 2 January 2022 which destroyed major parts of the parliamentary precinct, created a new predicament. Parliament had to move to a much smaller building, which cannot accommodate all its members and therefore a hybrid model is again used, which has limitations of its own. Normalisation of the parliamentary activities is therefore not yet possible.

The third factor—the role of civil society—was not discussed in depth. But we have seen that consultations with civil society organisations were limited in the absence of the National Disaster Management Advisory Forum. Organisations and business groups were prominent in challenging the constitutionality of the DMA, Command Council and several disaster regulations in court. The main conclusion in this respect revolves around public trust as part of a democratic context. Since the 2016 local government election, the ANC’s public support as governing party is in decline. The pandemic deepened it and a public trust deficit developed. Government members’ abuse of the regulations, their own violation of them, pandemic-related corruption and the lack of governance during the pandemic contributed to this distrust. It is directed mostly at the ANC and not at all the political parties, and therefore it cannot be generalised as a new democratic deficit.

The fourth factor—negative socio-economic consequences—as part of the substantive definition of democracy, was not discussed in detail. As a general observation, however, the pandemic definitely had a negative impact on South Africa’s socio-economic circumstances. Several relief measures were made available by Government, but they are not sufficient. It stimulated another debate on state-society relations and especially the socio-economic responsibilities of the state versus increasing prominence of the private sector. In future, it would have an impact on how South Africans perceive democracy.

The last factor—democratic consolidation and democratic quality—has wide-ranging implications. Freedom House’s assessment of South Africa during the pandemic as well as this discussion itself, are evidence that the consolidated state of this country’s democracy is not under threat. The quality of democracy, however, has been negatively affected by the pandemic. The limited role of Parliament, the opposition parties’ lack of opportunities, especially in Parliament, and the executive abuse of the pandemic powers all affected the democratic quality. On the other hand, although not discussed, the relative success of the local government elections in November 2021, should also be taken into account. For example, the election results included a change in government in a number of metropolitan councils, but its legitimacy was never challenged by electoral uncertainties. The same positive contribution to democratic quality came from civil society actions during the pandemic.

Finally, the pandemic and Government’s management of it, did affect the quality of democracy. But almost all the executive emergency functions during the pandemic have been reversed and Parliament is again more assertive. Given the experience of the state of disaster, it is unlikely that it will be deployed again in similar circumstances and therefore the flouting of new Health Act regulations happened. The pandemic’s psychological impact and public distrust in government are, however, not easy to determine or predict for the long-term.
